# A Survey of Neck Pain among Dentists of the Lebanese Community

**DOI:** 10.1155/2023/8528028

**Published:** 2023-03-23

**Authors:** Abed AlRaouf Kawtharani, Ali Msheik, Fadi Salman, Ali Haj Younes, Ammar Chemeisani

**Affiliations:** ^1^Internal Medicine, Lebanese University, Faculty of Medicine, Hadath, Beirut, Lebanon; ^2^Neurological Surgery, Lebanese University, Faculty of Medicine, Hadath, Lebanon; ^3^Neurological Surgery, Al Zahraa Hospital UMC, Jnah, Beirut, Lebanon; ^4^General Surgery, Lebanese University, Faculty of Medicine, Hadath, Beirut, Lebanon; ^5^Obstetrics and Gynecology, Lebanese University, Faculty of Medicine, Hadath, Beirut, Lebanon

## Abstract

**Results:**

The majority of participants were between the ages of 25 and 35, and the gender distribution of the demographic distribution was comparable. The prevalence of pain was 86.8% (97/342 dentists). NDI analysis showed that 65.7% had mild disability, 12.8% have a moderate disability, and 1% had severe disability. Bivariate analysis showed that pain was affected by age (*p*=0.013), orthodontist practices (*p*=0.031), regular exercise (*p* < 0.001), using vibrating instruments (*p* < 0.001), cervical flexion for better vision while working (*p* < 0.001), knowledge, and experience about ergonomic posture (*p* < 0.005). Multivariate analysis showed four predictors for pain: age (*p*=0.017), performing stretching exercises after finishing clinical practice (*p*=0.022), orthodontist specialty (*p*=0.029), and performing cervical flexion for better vision while working (*p*=0.004).

**Conclusion:**

This study showed that through the application of some strategies such as stretching, exercising, and being careful in using vibrating instruments, the dentist may be able to relieve the pain.

## 1. Introduction

The World Health Organization estimates that there are 1.71 billion musculoskeletal disorders worldwide. Multiple sclerosis (MSD), especially neck pain, is a significant occupational health hazard in dentistry [1]. These problems have been around for a while and are still prevalent now. In a 1990 poll of dentists, 72% said they occasionally or frequently experienced neck, shoulder, or head pain [2]. Dental professionals in Western nations reported 58.5% neck discomfort, 56.4% back pain, 43.1% shoulder pain, and 41.1% back pain in a 2018 poll [3]. Rheumatoid arthritis refers to a group of conditions that affect many components of the nervous system, including the muscles, tendons, cartilage, bones, blood, and appendages such as intervertebral discs [3]. A study in Iran revealed no statistically significant difference between the two sexes, in contrast to a study in Germany that found women reported more neck stress than males [4, 5]. Unknown gender prevalence exists. However, both male and female younger dentists see rheumatoid arthritis more frequently than older dentists [2]. It must be crucial to comprehend this idea in order to improve periodontal disease diagnosis and management, comprehend its causes, risk factors, and preventive measures, and raise knowledge of ergonomic concerns that impact a dentist's periodontal health and general well-being. Strong motor abilities are essential for dentistry's physically and intellectually demanding practice [3].

### 1.1. A Gap of Knowledge

Several characteristics and risk variables from our study have been used in other studies throughout the world to define observation or no glasses and the kind of treatment for neck and back pain. Previous research just sought to identify the frequency of neck and back issues among dentists. The final decision on the optimal conditions for dentists has not yet been made. Our research's objectives are to determine the most effective methods for dentists in Lebanon to utilize in preventing cervical cancer and instruct and interact with dental students.

### 1.2. Objectives

Several characteristics and risk variables from our study have been used in other studies throughout the world to define observation or no glasses and the kind of treatment for neck and back pain. Previous research just sought to identify the frequency of neck and back issues among dentists. The final decision on the optimal conditions for dentists has not yet been made. Our research's objectives are to determine the most effective methods for dentists in Lebanon to utilize in preventing cervical cancer and instruct and interact with dental students.

## 2. Materials and Methods

### 2.1. Study Design and Population

Between July 2022 and November 2022, the study used a cross-sectional survey to examine how neck pain affected a dentist's capacity for work and productivity. The number of dentists available in each region is the same as the ratio of dentists registered in Lebanon during the aforementioned time to dentists in the specified region. Although regional variances must be considered, Lebanon is a very small country, and regional distinctions are minimal.

### 2.2. Inclusion Criteria

Dentists who are currently working in Lebanon and have no history of whiplash injuries, whiplash-related diseases, or recent trauma were the study's participants. By selecting “Agree to accept” on the electronic permission form listed in Google forums, they have consented to take part in the study. The appendix illustrates the specifics of the consent (Table 1).

### 2.3. Exclusion Criteria

The study did not include retired dentists or dental students, dentists with spinal/musculoskeletal (MSK) conditions, or dentists who had recently experienced trauma or whiplash injury. In the process of gathering data and evaluating the variables, incomplete questionnaires were disregarded.

### 2.4. Sample Size

Based on the Slovin formula *n* = *N*/(1 + *N*.*e*^2^), where *n* is the “number of samples,” *N* is the “total population,” and *e* is the “error tolerance (level),” 340 participants are representative of the dentist population in Lebanon. “*N*” represents the number of dentists registered in the dentist syndicate in 2019 (2774 dentists). “*e*” represents the *p* value = 0.05. 353 dentists who practice in Lebanon as a whole took part in the survey, which is about the same size as the bare minimum, sample required (350 participants). There were 342 full surveys. Due to lacking data, 11 incomplete surveys were deleted and excluded.

### 2.5. Data Collection

An online validated survey was designed to be used in the data collection process. Its link was shared by social media and messages. Direct calls were also made with many dentists from all around Lebanon, and the link was sent after the call.

There will be three key sections to the questionnaire. Demographic information (gender, age) and the nature and duration of the dental practice—represented by the number of hours worked each week, general health status, and exercise habits—were all included in the first part. The work of Diaz-Caballero et al., which the authors validated, served as the basis for the second portion [6]. It asked questions based on Nordic questionnaires for the investigation of musculoskeletal symptoms [7] concerning ergonomic practices and the location of musculoskeletal pain. Additionally, a numeric pain rating scale from 0 (no pain) to 10 was used to gauge the degree of pain (worst pain possible). The Neck Disability Index (NDI), which is intended to determine how neck pain impacts the ability to handle daily tasks, was added in the third segment. The Likert scale has a 0–5 range, while the NDI has 10 items [8].  Table 2 shows the specifics of the questionnaire and the scoring methodology.

### 2.6. Ethical Considerations

This study was based on a cross-sectional survey. The RHUH IRB committee received the study proposal and the data collecting form (Rafic Hariri University Hospital). The IRB was approved, and a form seeking IRB approval was retrieved. The doctors' names were not requested, and top confidentiality was maintained. Everyone who took part got a study code. Each participant was told of the study's purpose, and an internet survey asked for his or her informed permission. All of the participants had been guaranteed that the data would stay private and that just the statistical elements would be looked at.

### 2.7. Data Management

A well-known technique for evaluating neck pain-related self-rated disability is the NDI. There are ten items in it. For each of the 10 items, scores are given between 0 and 5. Thus, the best possible score is 50. The reliability test found strong associations among the 10 components that make up the score NDI. The Alpha for Cronbach was 0.845. The score NDI was broken into 5 categories after item computation: No disability: NDI between 0 and 4; the NDI ranges from 5 to 14 for mild disability, 15 to 24 for moderate disability, and 25 to 34 for significant disability. Disability level 20: NDI of 34 or higher (Table 2).

### 2.8. Data Analysis

IBM SPSS version 25 was used for all statistical analyses. The variables were reported according to their kind in a descriptive analysis that was conducted. The frequency and percentage were used to present the categorical variables (for example, gender and specialty). For the continuous variables, the frequency, mean, median, and standard deviation were shown (for example, Pain Score and NDI). The Chi-square test and Fisher exact test were used in bivariate analysis to examine the connection between two nominal variables (for example, pain and gender). The odds ratio is denoted by “OR.” The correlation between one continuous variable (not normally distributed) and one nominal variable was examined using the Student's *t*-test (for example, pain and NDI). To forecast the likelihood that dentists would experience work-related musculoskeletal pain, multiple logistic regression models were used. All hypotheses were tested at a significance level of 0.05 (using an alpha error of 5%).

### 2.9. Data Bias

Dentists were approached one-on-one to talk about questions of misinterpretation of either of the questionnaire's options and to consider any potential bias. The authors walked dentists with a French education through the questionnaire to make sure they understood their options. After trying unsuccessfully to reach the subject, missing information or incomplete questionnaires were eliminated.

## 3. Results

### 3.1. Demographic Characteristics

201 (58.8%) of the 342 dentists were men and 141 (41.2%) were women. 51.2% of participants were between the ages of 25 and 35, 30.4% were between the ages of 36 and 45, 10.5% were between the ages of 46 and 56, and 7.9% were over the age of 56 (Table 3).

Among the 342 dentists, 145 (42%) engaged in regular exercise, while 197 (58%) did not. During dental practice, out of 342 dentists, 85.4% were able to change their posture, whether they were sitting or standing; 64.9% frequently changed positions; 32.7% stretched after finishing clinical practice; 47.4% were handling instruments within hand reach without making strenuous movements; 91.2% performed torsions or cervical flexions to improve vision when working in the oral cavity; and 26.0% crossed their legs (Table 3).

### 3.2. Pain-Related Characteristics

**Figure 1:**
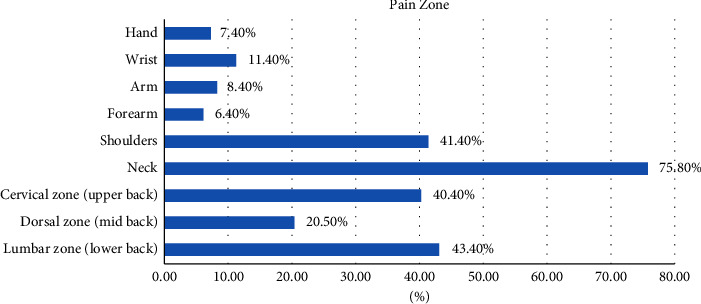
Pain zone distribution.

**Figure 2:**
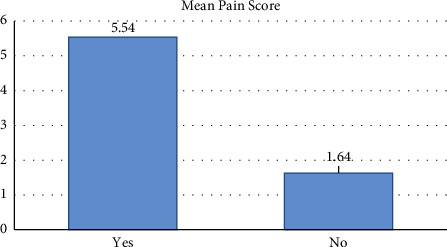
Pain score as a function of the existence of pain; *p* < 0.001.

Out of 342 dentists, 297 (86.8%) confirmed having muscular pain due to dental practice, whereas only 45 (13.2%) did not approve of having any pain. Out of 342 dentists, 153 (44.7%) experienced pain using vibrating instruments, 297 (86.8%) performed cervical flexion for better vision while working, and 254 (74.3%) were familiar with the ergonomic posture to perform clinical procedures in your dental practice. The most frequent activity causing muscular discomfort was related to endodontics (69.4%) out of 297 dentists who verified having pain, followed by surgery (41.4%), restorative surgery (30.6%), and the activities related to endodontics (15.5%). Out of 297 dentists who admitted to experiencing pain, the neck (75.8%), lumbar zone (43.4%), shoulders (41.4%), and dorsal zone (20.5%) were the top five pain zones (Figure 1). A pain score, from 0 to 10, was used to assess the level of pain in dentists. The correlation between the presence of pain and the pain score was statistically different (*p* 0.001). When compared to dentists who confirmed they were pain-free (mean pain score = 1.64), dentists who confirmed they were in pain had a higher mean score (5.54) (Figure 2).

### 3.3. Neck Disability Index

To measure the degree of neck pain in dentists, the Neck Disability Index was utilized. Table 3 displays the outcomes for the 10 questions. The findings indicated minimal levels of pain severity, and Table 3 lists the 10 items' most frequent outcomes. The Neck Disability Index has four categories. Out of 297 dentists, 61 (20.5%) were handicapped, 195 (65.7%) had a light impairment, 38 (12.8%) had a moderate impairment, and only three (1%) had a severe impairment. The relationship between the NDI and the occurrence of pain was statistically different (*p* <  0.001). When compared to dentists who affirmed they were pain-free (mean NDI = 2.69), dentists who confirmed they were in pain had a higher mean score (mean NDI = 8.6) (Table 4).

### 3.4. Factors Affecting Pain

The gender of the dentists did not statistically differ from the frequency of discomfort (*p*=0.614). 58.2% of the 297 dentists who admitted to experiencing discomfort were men, and 41.8% were women (Table 5). The dentists' age and the occurrence of discomfort differed statistically significantly (*p*=0.013). Dentists who reported experiencing pain are older than dentists who reported experiencing no discomfort. 52.2% of the 297 dentists who reported experiencing discomfort were older than 35, compared to 73.3% of dentists who reported experiencing no pain (Table 5).

Among the 6 specialties, only 1 specialty, orthodontist, was shown a statistical difference in the function of the pain occurrence (Table 6). Only 5.1% of dentists who confirmed having pain were orthodontists compared to 13.3% (OR: 0.346; CI: 0.127–2.991) of those who confirmed having no pain. The fact of being a dentist and not practicing orthodontics increase the risk of pain by 0.346 times.

The relationship between the frequency of pain and the number of working hours was not statistically different (*p*=0.168). Of the 297 dentists who admitted to experiencing pain, 34% worked 15‒30 hours per week, 53.2% worked 31‒40 hours per week, and 12.8% worked 41‒50 hours per week (Table 6). The presence of pain and the history of spine disease did not differ statistically significantly (*p*=0.654). 4 (1.3%) of the 297 dentists who reported pain had a congenital spinal disease, 9 (3%) had had spine surgery, and 284 (95.6%) had no prior history of spine disease (Table 6).

Physical activity and the occurrence of pain differed statistically significantly (*p* < 0.001). According to the findings, 64.3% of dentists who admitted to experiencing discomfort did not engage in physical exercise, as opposed to 13.3% of dentists who admitted to experiencing no pain. Not Physical exercise increases the likelihood of pain by 0.085 times (Table 6). The difference between experiencing pain and doing so while utilizing vibrating instruments was statistically significant (*p*  < 0.001). According to the findings, vibrating devices were utilized by 50.8% of dentists who acknowledged feeling discomfort, as opposed to 4.4% of dentists who acknowledged feeling no pain. The probability of experiencing pain is increased by 22.236 times by utilizing vibrating equipment (Table 6).

Between experiencing pain and flexing the neck for better working-related vision, there was a statistically significant difference (*p* <  0.001). According to the findings, 89.2% of dentists who admitted to experiencing pain engaged in cervical flexion for improved eyesight while working, as opposed to 71.1% of dentists who admitted to experiencing no pain. Cervical flexion for enhanced eyesight really raises pain risk by 3.364 times (Table 7). Knowing the ergonomic posture to use when doing clinical operations in a dental office and the occurrence of pain were statistically different (*p*=0.002). The findings revealed that 71.4% of dentists who admitted to experiencing discomfort were conversant in ergonomic posture, as opposed to 93.3% of dentists who admitted to experiencing no pain. Utilizing an ergonomic posture when doing clinical tasks in a dental office reduces the chance of pain by 0.178 times (Table 7).

During dental practice, there was a statistically significant difference between experiencing discomfort and being able to adjust posture, seating, or standing (*p*=0.023). The findings revealed that 83.8% of dentists who admitted to experiencing discomfort during dental practice were able to adjust their work position, as opposed to 95.6% of dentists who admitted to experiencing no pain. The likelihood of pain is reduced by altering work posture (Table 8). Changing positions during clinical practice (*p*=0.108), stretching after clinical practice (*p*=0.073), handling instruments within hand reach without making strenuous movements (*p*=0.133), performing torsions or cervical flexions to improve vision when working in the oral cavity (*p*=0.246), and crossing leg over leg did not statistically differ from the occurrence of pain (*p* > 0.05) (Table 8).

Among the 342 dentists who reported pain, 83.8% were able to adjust their posture while sitting or standing, 63.3% frequently switched positions, 31% stretched after finishing clinical practice, 47.4% handled instruments within easy reach without making strenuous motions, 91.9% performed torsions or cervical flexions to improve vision when working in the oral cavity, and 26.9% crossed their legs (Table 8).

Finally, a multivariate analysis was enrolled to test the factors affecting pain in dentists. 4 factors affecting pain: age (*p*=0.017), performing stretching exercises after finishing clinical practice (*p*=0.022), orthodontist specialty (*p*=0.029), and performing cervical flexion for better vision while working (*p*=0.004) (Table 9). The risk of pain increased with age (OR = 1.765), when not performing stretching exercises after finishing clinical practice (OR = 0.451), when practicing dental activities other than orthodentistry (OR = 0.284), and when performing cervical flexion for better vision while working (OR = 3.185).

## 4. Discussion

342 dentists in Lebanon were interviewed for this study; 58.8% of them were men and 41.2% were women. The respondents' occupational characteristics were diverse. As would be predicted, more dentists (86.8% vs. 13.2%) reported experiencing muscle soreness than those who did not. Many studies have been conducted to determine the primary pain regions in dentists. This study found that neck pain is the most prevalent (75.8%), followed by lumbar pain (43.4%), cervical pain (40.4%), and shoulder pain (40.4%). With 236 dentists reporting varying degrees of a neck disability, we discovered a substantial association (*p* <  0.001) between the presence of discomfort and the Neck Disability Index (NDI) in this study. The study by Aghahi, Darabi, and Hashemipour found a connection between discomfort and musculoskeletal diseases and sitting position, work environment, and dental chair [10]. Ajwa et al.'s research also revealed that age, academic success, and exercise were risk factors for musculoskeletal issues [11]. Age but not gender were linked with MSDs in our study (*p*=0.614). Regarding specialty, the findings showed that when a dentist's area of expertise was something other than orthodontics, the incidence of pain increased by 0.346. Working hours had no correlation with musculoskeletal pain (*p*=0.168). Also, there was no danger associated with the spinal posture (*p*=0.654). *p* values are connected to MSDs for each of the following factors: physical activity, vibrating machinery, the flexibility of the neck for better visibility at work, and knowledge of the ergonomic position to carry out clinical operations in dental practices. Our current study demonstrated no correlation between working hours per week and musculoskeletal pain (*p*=0.168), despite the fact that dentists routinely put in between 41 and 50 hours per week. The number of hours worked each day and hand stiffness, however, were found to be significantly correlated by Sheikh et al. (2011) (*p*=0.018) [6]. Our second aim leads us to the conclusion that, while working hours do not appear to have an impact, the ergonomic position is associated with the presence or absence of muscle difficulties.

Finding out how stretching exercises improve human performance was the third goal of this study. The results show that less than half of the sample size, or 31% of dentists in Lebanon, conducted these exercises following a clinical practice. Four characteristics were shown to have an impact on oral musculoskeletal discomfort in the final multivariate analysis of these results: age (*p*=0.017), stretching exercises after use (*p*=0.022), being an orthodontist (*p*=0.029), and use of cervical tilt (*p*=0.004).

Parallel to this, other research looked for variables that might affect dental pain. In one study, the number of patients treated daily and the dentist's weight were linked to muscular discomfort, although age did not appear to have any bearing [6].

The prevalence of musculoskeletal (MSK) disease was found to be 90.2% in a cross-sectional study of 184 dentists in Saudi Arabia, with the following indicators of MSK disease: age (OR 1.23; 95% CI 1.00‒1.50), gender (OR 2.52; 95% CI 1.12‒5.68), time spent by the dentist with patients (OR 0.28; 95% CI 0.14‒0.54), and years of experience (*p* <  0.05).

These findings suggest that musculoskeletal pain is a significant burden for dentists, who must move their hands and wrists correctly while working and sitting in particular positions, which might be challenging if they have musculoskeletal problems. It might be unpleasant. The risk factors that contribute to the development of musculoskeletal pain and MSD, as well as pain-reduction techniques such as stretching and exercise, are better understood.

Working unpleasant or long hours was the risk factor that was most clear [11]. The primary causes of neck pain are time spent working and studying, workload, and physical circumstances at work [12]. Significant risk factors were workplace characteristics, perceived job demands, the harmony between effort and reward, and colleague support.

Psychological factors and neck and back issues are strongly correlated [13, 14]. According to research from the Chinese Mental Health Survey [15], people with various forms of mental illness are more likely to experience persistent back or neck pain and mood disorders. A prospective study has revealed that psychological factors are connected to the incidence and severity of disease (e.g., acute, subacute, and chronic). Neck pain has been demonstrated to be significantly influenced by stress, depressive symptoms, anxiety, mood, and emotional states, as well as by behaviors that are associated with pain. There is not much proof of personality, though. In general, pain processing in the spinal cord, brainstem, or cerebral cortex can be affected by stress, pain results, depressive symptoms, lack of sleep, and alcohol, leading to telekinetic hyperalgesia. More study is required to comprehend how lifestyle factors affect central pain processes in nontraumatic neck pain due to these cognitive, emotional, and behavioral components. An overview of how sickness affects the four psychological domains of cognition, emotions, social interactions, and conduct is provided. The cognitive element is the first and consists of attitudes, beliefs, and perceptions about the disease, incapacity, and perceived health. The three main elements of the emotional dimension, which is the second theme, are sadness, anxiety, and depression. Finally, there is a social element. Although the data are sketchy, back and neck pain seem to be linked to issues at both work and home. Lastly, a behavioral domain has also been identified [13, 16–19], which mainly consists of activity patterns, pain behavior, and adaptation. Vulnerability and stress are closely associated [13, 14]. While under a lot of stress, neck pain is more likely to develop [17, 20]. Adolescents with neck pain had significantly greater stress indices than adolescents without neck pain, according to at least two studies of medium methodological quality. Moreover, chronic or persistent internal stress is linked to an increased risk of reporting neck pain [21]. Stress may have an impact on altered central pain processing at the spinal cord, brainstem, or cortex levels. Pain sensitivity is higher in people who have distal hyperalgesia [22, 23]. Stress also plays a role in mediating the link between pain and impairment [24, 25].

Disability and anxiety are linked to a variety of chronic disorders, including neck discomfort [13, 14, 26, 27]. There is evidence that oral discomfort and anxiety can coexist [27–29]. Researchers measured trait and state anxiety in teenagers with and without neck pain using two distinct assessment techniques, and they discovered that adolescents with neck pain had greater trait and state anxiety levels than adolescents without neck pain. Also, it was discovered that anxiety disorders and neck pain were the second most frequent comorbidities, with specific phobias being the main issue among patients with these diseases.

Lower pressure pain thresholds are linked to higher levels of anxiety (PPT). PPTs have been linked in studies to neck pain severity, duration, recurrence, and disability [30]. Anxiety has been linked to increased levels of anxiety in people with neck pain [28], and anxiety worsens to pain and impairment [24]. The results are inconsistent in certain ways, though. Anxiety, for instance, does not mediate pain and impairment, according to studies on psychological distress variables (such as stress, anxiety, and depression) [25]. A thorough examination of the neck and other characteristics utilized to treat MSK discomfort is given by Kazeminasab et al. in their study from [31].

### 4.1. Impact of the Study

In the long-term, dentists are seriously at risk from musculoskeletal conditions. This study contributes to the corpus of literature by providing comprehensive knowledge about musculoskeletal pain, its effects, risk factors, treatment, and preventative strategies. According to our research, a number of factors may contribute to increased musculoskeletal pain, and a number of techniques may be effective in preventing or lessening it. This study offers a chance to look into the causes of musculoskeletal disorders and give dentists the proper training to understand how ergonomic factors affect their health.

### 4.2. Study Limitations

This study had certain restrictions. The sample size was quite small, to begin with, when compared to the total number of dentists in Lebanon who are licensed to practice, which, according to records maintained by the Lebanese Dental Association, is 2774 dentists. This could have reduced the study's statistical power and prevented the identification of several key risk factors for musculoskeletal illnesses. Second, the percentages between the dentists' specialties were highly different from one another, which could have tampered with the results. Last but not the least, this study focuses on self-reporting, which could add some confounding factors and bias due to language.

### 4.3. Study Perspectives

Future studies should consider conducting broad polls to learn more about the risk factors related to dentists developing musculoskeletal pain, especially in view of the fact that several studies seem to yield conflicting findings. As a result, a larger sample size should be considered. Besides ergonomics and sociodemographic characteristics, other factors that may affect the development of sickness and physical discomfort include psychological factors. Finally, observational studies should be taken into account to reduce the bias caused by self-reporting.

## 5. Conclusion

This study's main goal was to find out how neck pain affected the productivity and efficiency of dental offices. It additionally aimed to comprehend the reasons and remedies for musculoskeletal pain. The impact of extending work capacity as well as the influence of duration—represented by the number of hours worked each week and the position adopted during clinical practice—were both heavily emphasized. In this study, 342 dentists who practice in Lebanon were analyzed. According to our research, dentists can lessen the discomfort by using certain procedures including stretching, exercise, and judicious vibration machine use. Large-scale investigations are required in addition to dental monitoring (if possible) and research on treatment and preventative strategies. Studying the answer is crucial after comprehending the nature of the issue. Otherwise, all you do would be in vain.

## Figures and Tables

**Table 1:** Details of the electronic consent.

Confirmation statements	I confirm that I have read and understood all the information related to the above study	
	I had the opportunity to ask questions, which have been answered fully	
	I understand that my participation in this study is voluntary and I have the right to withdraw from the above study at any point without giving any reason, and without my medical care or legal rights being affected	
	I acknowledge that my name will not be used at any point in the above study	

Declaration of consent to participate	I agree to participate	Yes
		No

**Table 2:** Questionnaire sections and questions per each section with the corresponding choices; first introduced in 1996 [6, 9].

Questionnaire section	Question per section	Choices	
Demographic data	Gender	Male	
		Female	
	Age	25–35 years	
		36–45 years	
		46–56 years	
		>56 years	
	Type of practice	Governmental	
		Private	
		Both	
	Specialty	General dentist	
		Periodontist endodontist	
		Prosthodontist	
		Pedodontist	
		Maxillofacial surgeon	
		Orthodontist	
	Hours of work per week	15–30	
		31–40	
		41–50	
	Regular exercise	Yes	
		No	
	Experiencing pain using vibrating instruments	Yes	
		No	
	Cervical flexion for better vision while working	Yes	
		No	
	Do you have any history of those mentioned below? If yes, please select which one (s)	Congenital spinal disease	
		Spine trauma	
		Spine surgery	
		None	

Ergonomics	Do you have muscular pain due to dental practice?	Yes	
		No	
	Are you familiar with the ergonomic posture to perform clinical procedures in your dental practice?	Yes	
		No	
	Which activities of your clinical practice produce muscular pain? Mark the main activities	Surgery	
		Endodontics periodontics	
		Restorative	
	Reference the previous question: mark in which zone you feel the pain	Lumbar zone (lower back)	
		Dorsal zone (mid-back)	
		Cervical zone (upper back)	
		Neck	
		Shoulders	
		Forearm	
		Arm	
		Wrist	
		Hand	
		Other:	
	Are you able to change your work posture, seating or standing, during your practice?	Yes	
		No	
	Do you frequently change positions during your clinical practice?	Yes	
		No	
	After finishing clinical practice, do you perform stretching exercises?	Yes	
		No	
	Are the instruments within hand reach without making strenuous movements?	Yes	
		No	
	Do you perform torsions or cervical flexions to improve vision when working in the oral cavity?	Yes	
		No	
	Do you cross your legs while working?	Yes	
		No	
	On a scale of 0 to 10, what is the intensity of the neck pain?	No pain	
		0	
		1	
		2	
		3	
		4	
		5	
		6	
		7	
		8	
		9	
		10	
		Worst pain possible	

Neck Disability Index (NDI)	NDI subsection	Response	Score
	NDI 1: pain intensity	I have no neck pain now	0
		The pain is very mild now	1
		The pain is moderate now	2
		The pain is severe now	3
		The pain is very severe now	4
		The pain is the worst imaginable now	5
	NDI 2: personal care	I can look after myself normally without causing extra neck pain	0
		I can look after myself normally, but it causes extra neck pain	1
		It is painful to look after myself, and I am slow and careful	2
		I need some help but manage most of my personal care	3
		I need help every day in most aspects of self-care	4
		I do not get dressed, I wash with difficulty, and stay in bed	5
	NDI 3: lifting	I can lift heavy weights without causing extra neck pain	0
		I can lift heavy weights, but it gives me extra neck pain	1
		Neck pain prevents me from lifting heavy weights off the floor but I can manage if items are conveniently positioned, i.e., on a table	2
		Neck pain prevents me from lifting heavy weights, but I can manage light weights if they are conveniently positioned	3
		I can lift only very light weights	4
		I cannot lift or carry anything at all	5
	NDI 4: reading	I can read as much as I want with no neck pain	0
		I can read as much as I want with slight neck pain	1
		I can read as much as I want with moderate neck pain	2
		I cannot read as much as I want because of moderate neck pain	3
		I cannot read as much as I want because of severe neck pain	4
		I cannot read at all	5
	NDI 5: headaches	I have no headaches at all	0
		I have slight headaches that come infrequently	1
		I have moderate headaches that come infrequently	2
		I have moderate headaches that come frequently	3
		I have severe headaches that come frequently	4
		I have headaches almost all the time	5
	NDI 6: concentration	I can concentrate fully without difficulty	0
		I can concentrate fully with sight difficulty	1
		I have a fair degree of difficulty concentrating	2
		I have a lot of difficulty concentrating	3
		I have a great deal of difficulty concentrating	4
		I cannot concentrate at all	5
	NDI 7: work	I can do as much work as I want	0
		I can only do my usual work, but no more	1
		I can do most of my usual work, but no more	2
		I cannot do my usual work	3
		I can hardly do any work at all	4
		I cannot do any work at all	5
	NDI 8: driving	I can drive as long as I want without neck pain	0
		I can drive as long as I want with only slight neck pain	1
		I can drive as long as I want with moderate neck pain	2
		I cannot drive as long as I want because of moderate neck pain	3
		I can hardly drive at all because of severe neck pain	4
		I cannot drive my car at all because of neck pain	5
	NDI 9: sleeping	I have no trouble sleeping	0
		My sleep is slightly disturbed for less than 1 hour	1
		My sleep is mildly disturbed for up to 1-2 hours	2
		My sleep is moderately disturbed for up to 2-3 hours	3
		My sleep is greatly disturbed for up to 3–5 hours	4
		My sleep is completely disturbed for up to 5–7 hours	5
	NDI 10: recreation	I am able to engage in all my recreational activities with no neck pain at all	0
		I am able to engage in all my recreational activities with some neck pain	1
		I am able to engage in most, but not all of my recreational activities because of the pain in my neck	2
		I am able to engage in a few of my recreational activities because of the pain in my neck	3
		I can hardly do recreational activities due to neck pain	4
		I cannot do any recreational activities due to neck pain	5

**Table 3:** Demographic characteristics of dentists.

Factor		Answer	Frequency	Percentage
Gender		Male	201	58.8
		Female	141	41.2

Age		25–35 years	175	51.2
		36–45 years	104	30.4
		46–56 years	36	10.5
		>56 years	27	7.9

Type of practice		Private	234	68.4
		Governmental	8	2.3
		Both	100	29.2

Specialty		General dentist	207	60.5
		Endodontist	44	12.9
		Prosthodontist	21	6.1
		Pedodontist	41	12.0
		Maxillofacial surgeon	8	2.3
		Orthodontist	21	6.1

Working hours		15–30 hours/week	112	32.7
		31–40 hours/week	182	53.2
		41–50 hours/week	48	14.0

Assessment questions	Are you able to change your work posture, seating or standing, during your practice?	No	50	14.6%
		Yes	292	85.4%
	Do you frequently change positions during your clinical practice?	No	120	35.1%
		Yes	222	64.9%
	After finishing clinical practice, do you perform stretching exercises?	No	230	67.3%
		Yes	112	32.7%
	Are the instruments within hand reach without making strenuous movements?	No	180	52.6%
		Yes	162	47.4%
	Do you perform torsions or cervical flexions to improve vision when working in the oral cavity?	No	30	8.8%
		Yes	312	91.2%
	Do you cross your legs while working?	No	253	74.0%
		Yes	89	26.0%

NDI	Pain intensity	I have no neck pain at the moment	121	35.4
		The pain is very mild at the moment	130	38.0
		The pain is moderate at the moment	80	23.4
		The pain is fairly severe at the moment	6	1.8
		The pain is very severe at the moment	5	1.5
	Personal care	I can look after myself normally without causing extra neck pain	180	52.6
		I can look after myself normally, but it causes extra neck pain	143	41.8
		It is painful to look after myself, and I am slow and careful	13	3.8
		I need some help but manage most of my personal care	5	1.5
		I need help every day in most aspects of self-care	1	0.3
	Lifting	I can lift heavy weights without causing extra neck pain	159	46.5
		I can lift heavy weights, but it gives me extra neck pain	118	34.5
		Neck pain prevents me from lifting heavy weights off the floor but I can manage if items are conveniently positioned	47	13.7
		Neck pain prevents me from lifting heavy weights, but I can manage light weights if they are conveniently positioned	5	1.5
		I can lift only very light weights	12	3.5
		I cannot lift or carry anything at all	1	0.3
	Reading	I can read as much as I want with no neck pain	115	33.6
		I can read as much as I want with slight neck pain	170	49.7
		I can read as much as I want with moderate neck pain	48	14.0
		I cannot read as much as I want because of moderate neck pain	9	2.6
	Headaches	I have no headaches at all	113	33.0
		I have slight headaches that come infrequently	161	47.1
		I have moderate headaches that come infrequently	50	14.6
		I have moderate headaches that come frequently	11	3.2
		I have severe headaches that come frequently	7	2.0
	Concentration	I can concentrate fully without difficulty	218	63.7
		I can concentrate fully with sight difficulty	83	24.3
		I have a fair degree of difficulty concentrating	37	10.8
		I have lot of difficulty concentrating	3	0.9
		I have a great deal of difficulty concentrating	1	0.3
	Work	I can do as much work as I want	128	37.4
		I can only do my usual work, but no more	205	59.9
		I can do most of my usual work, but no more	3	0.9
		I cannot do any work at all	6	1.8
	Driving	I can drive as long as I want without neck pain	8	2.3
		I can drive as long as I want with only slight neck pain	260	76.0
		I can drive as long as I want with moderate neck pain	74	21.6
	Sleeping	I have no trouble sleeping	204	59.6
		My sleep is slightly disturbed for less than 1 hour	92	26.9
		My sleep is mildly disturbed for up to 1-2 hours	34	9.9
		My sleep is moderately disturbed for up to 2-3 hours	5	1.5
		My sleep is greatly disturbed for up to 3–5 hours	3	0.9
		My sleep is completely disturbed for up to 5–7 hours	4	1.2
	Recreation	I am able to engage in all my recreational activities with no neck pain at all	150	43.9
		I am able to engage in all my recreational activities with some neck pain	146	42.7
		I am able to engage in most, but not all of my recreational activities because of pain in my neck	40	11.7
		I am able to engage in a few of my recreational activities because of pain in my neck	6	1.8

**Table 4:** NDI in the function of the existence of pain.

Pain	*N*	Mean	Std. deviation	Minimum	Maximum	*p* value
No	45	2.69	2.67	1.00	14.00	<0.0001^*∗*^
Yes	297	8.62	4.97	1.00	26.00	
Total	342	7.84	5.14	1.00	26.00	

**Table 5:** Correlation between muscle pain and demographic characteristics.

		Muscular pain due to dental practice		*p* value
		No	Yes	
Gender	Male	28	173	0.614^*∗*^
		62.2%	58.2%	
	Female	17	124	
		37.8%	41.8%	

Age	25–35 years	33	142	0.013^*∗*^
		73.3%	47.8%	
	36–45 years	6	98	
		13.3%	33.0%	
	46–56 years	3	33	
		6.7%	11.1%	
	>56 years	3	24	
		6.7%	8.1%	

Hours of work per week	15–30 hours/week	11	101	0.168
		24.4%	34.0%	
	31–40 hours/week	24	158	
		53.3%	53.2%	
	41–50 hours/week	10	38	
		22.2%	12.8%	

History of spine disease	Congenital spinal disease	0	4	0.654
		0.0%	1.3%	
	Spine surgery	2	9	
		4.4%	3.0%	
	None	43	284	
		95.6%	95.6%	

^*∗*^Chi-square test.

**Table 6:** Correlation between muscle pain and specialty.

		Muscular pain due to dental practice		*p* value	OR	95% Cl
		No	Yes			
General dentist	No	18	117	0.938	1.026	0.541–1.946
		40.0%	39.4%			
	Yes	27	180			
		60.0%	60.6%			

Endodontist	No	43	255	0.070	3.541	0.827–15.170
		95.6%	85.9%			
	Yes	2	42			
		4.4%	14.1%			

Prosthodontist	No	40	281	0.136	0.456	0.158–1.311
		88.9%	94.6%			
	Yes	5	16			
		11.1%	5.4%			

Pedodontist	No	41	260	0.492	1.459	0.494–4.308
		91.1%	87.5%			
	Yes	4	37			
		8.9%	12.5%			

Maxillofacial surgeon	No	44	290	0.956	1.062	0.128–8.841
		97.8%	97.6%			
	Yes	1	7			
		2.2%	2.4%			

Orthodontist	No	39	282	0.031	0.346	0.127–2.991
		86.7%	94.9%			
	Yes	6	15			
		13.3%	5.1%			

^*∗*^OR: odds ratio. ^*∗*^Chi-square test.

**Table 7:** Correlation between muscle pain and pain-related characteristics.

		Muscular pain due to dental practice		*p* value	OR	95% CI
		No	Yes			
Regular exercise	No	6	191	<0.001	0.085	0.035–0.208
		13.3%	64.3%			
	Yes	39	106			
		86.7%	35.7%			

Experiencing pain using vibrating instruments	No	43	146	<0.001	22.236	5.290–93.469
		95.6%	49.2%			
	Yes	2	151			
		4.4%	50.8%			

Cervical flexion for better vision while working	No	13	32	<0.001	3.364	1.602–7.063
		28.9%	10.8%			
	Yes	32	265			
		71.1%	89.2%			

Are you familiar with the ergonomic posture to perform clinical procedures in your dental practice?	No	3	85	0.002	0.178	0.054–0.590
		6.7%	28.6%			
	Yes	42	212			
		93.3%	71.4%			

**Table 8:** Correlation between muscle pain and position-related characteristics.

		Muscular pain due to dental practice		Total	*p* value
		No	Yes		
Are you able to change your work posture, seating or standing, during your practice?	No	2	48	50	0.023
		4.4%	16.2%	14.6%	
	Yes	43	249	292	
		95.6%	83.8%	85.4%	

Do you frequently change positions during your clinical practice?	No	11	109	120	0.108
		24.4%	36.7%	35.1%	
	Yes	34	188	222	
		75.6%	63.3%	64.9%	

After finishing clinical practice, do you perform stretching exercises?	No	25	205	230	0.073
		55.6%	69.0%	67.3%	
	Yes	20	92	112	
		44.4%	31.0%	32.7%	

Are the instruments within hand reach without making strenuous movements?	No	19	161	180	0.133
		42.2%	54.2%	52.6%	
	Yes	26	136	162	
		57.8%	45.8%	47.4%	

Do you perform torsions or cervical flexions to improve vision when working in the oral cavity?	No	6	24	30	0.246
		13.3%	8.1%	8.8%	
	Yes	39	273	312	
		86.7%	91.9%	91.2%	

Do you cross your legs while working?	No	36	217	253	0.323
		80.0%	73.1%	74.0%	
	Yes	9	80	89	
		20.0%	26.9%	26.0%	

^*∗*^Chi-square test.

**Table 9:** Binary logistics analysis for factors affecting the pain.

	*B*	S.E	*p* value	OR
Age	0.568	0.238	0.017	1.765
After finishing clinical practice, do you perform stretching exercises?	−0.796	0.349	0.022	0.451
Orthodontist	−1.257	0.576	0.029	0.284
Cervical flexions for better vision while working	1.158	0.399	0.004	3.185
Constant	0.446	0.525	0.396	1.561

## Data Availability

The data used to support the findings of this study are available from the corresponding author upon request.

## Appendix

### A. Electronic Informed Consent

#### A.1. Briefing

We are medical students at the Faculty of Medicine at the Lebanese University, and we are doing a study. Our goal is to study neck pain that dentists in Lebanon suffer from related to different characteristics, and we appreciate it if we can take your consent to answer the below questions to help us gather data for our study. Thank you for your time and collaboration.

#### A.2. Name of Principal Investigators

Dr. Abed Al Raouf Kawtharani, Dr. Fadi Salman, Dr. Ali Hajj Younes, Dr. Ali Msheik, and Dr. Ammar Chmeiseni.

#### A.3. Consent Form

The consent is detailed in Table 1.
